# Safe insertion of Veress needle for the induction of pneumoperitoneum: a technical note

**DOI:** 10.1093/jscr/rjad311

**Published:** 2023-06-01

**Authors:** Fahreyar Alam, Rebecca Badminton, Filip Tsvetkov, Zulfiqar Hanif, Richard Payne

**Affiliations:** General Surgery, Great Western Hospital, Swindon, UK; General Surgery, Great Western Hospital, Swindon, UK; General Surgery, Great Western Hospital, Swindon, UK; General Surgery, Great Western Hospital, Swindon, UK; General Surgery, Great Western Hospital, Swindon, UK

## Abstract

Techniques for the induction of pneumoperitoneum for laparoscopic surgery remain varied as complication risk remains with all techniques. Veress needle is used for the induction of pneumoperitoneum as a technique of preference or when an open technique is considered challenging as in obese patients. We present a novel safe technique for insertion of Veress needle by measuring the depth of the anterior abdominal wall prior to insertion. Accurate measurements help in the safe insertion of the Veress needle for inducing pneumoperitoneum and hence reduce the incidence of intra-abdominal injuries.

## INTRODUCTION

Gaining access into the abdominal cavity is a prerequisite for minimally invasive surgical procedures. However, the insertion of the Veress needle and trocars necessary for the induction of the pneumoperitoneum is associated with complications such as retroperitoneal vascular injury, intestinal perforation, wound herniation, abdominal wall haematoma and wound infection [1]. Around half of these complications are sustained during the entry into the abdomen [2–4]. Vascular injury because of the close proximity of the anterior abdominal wall to retroperitoneal structures remains relatively rare; however, it has a reported mortality of 15% [5].

The two commonly used techniques to achieve pneumoperitoneum and laparoscopy entry are the open and closed entry techniques. Closed entry can further be divided into Veress entry, which involves the insertion of a Veress needle at the level of the umbilicus and entry via direct insertion of a primary trocar without previous establishment of the pneumoperitoneum. Open entry also termed Hasson entry involves the incision and dissection through the abdominal wall via the umbilicus with subsequent insertion of a blunt-tipped catheter and creation of pneumoperitoneum through the trocar. Over the past several decades, there have been numerous techniques introduced to eliminate the risks associated with induction of the pneumoperitoneum; however, no single technique has been proven to eliminate complications. Indeed, while inserting the Veress needle via closed entry visceral or vascular injury may occur, as well as omental emphysema, whereas superficial entry may cause subcutaneous emphysema. Here, we describe a novel closed entry technique for the insertion of Veress needle through the approximate measurement of the anterior abdominal wall and subsequent induction of pneumoperitoneum.

## STEPS

**Figure 1:**
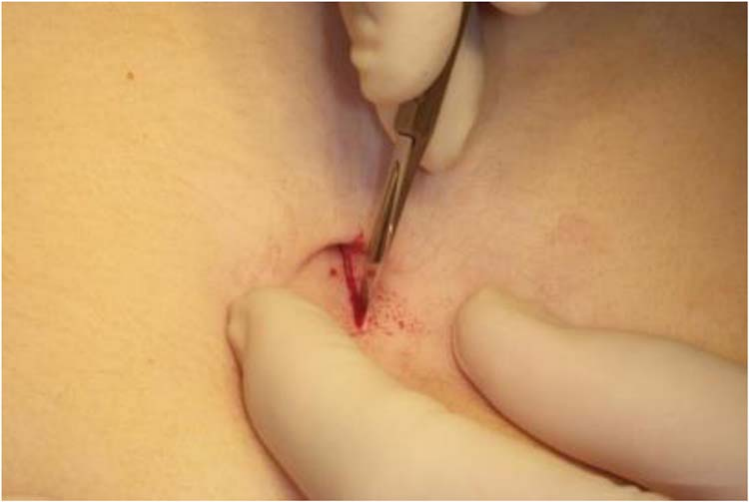
Image of incision site for surgical technique.

**Figure 2:**
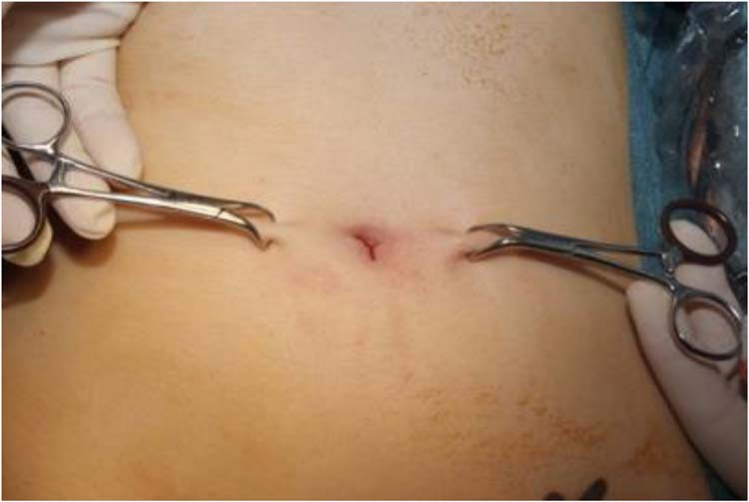
Image of towel clips elevating skin.

**Figure 3:**
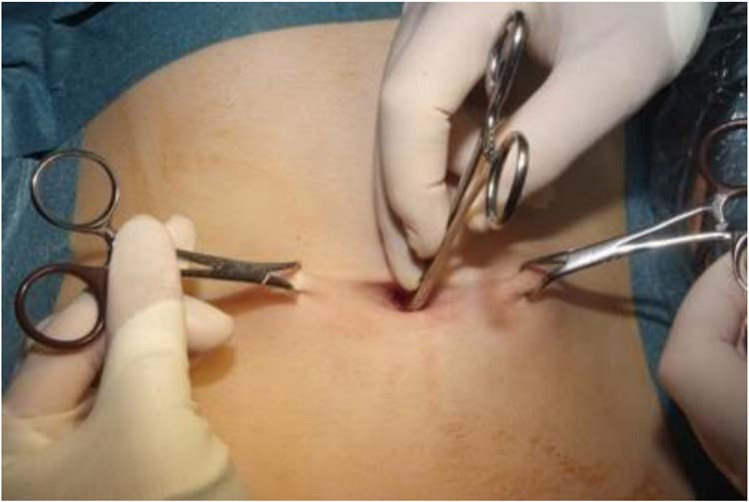
Image of the depth of skin measurement with artery forceps.

**Figure 4:**
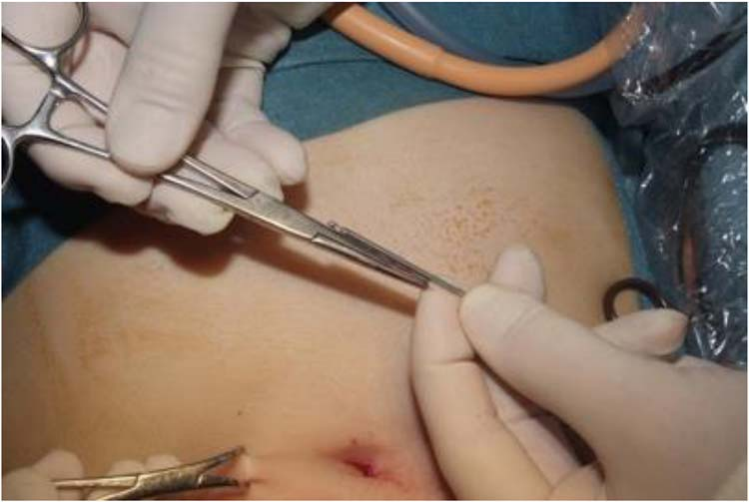
Image of the estimation of this depth with the Veress needle.

**Figure 5:**
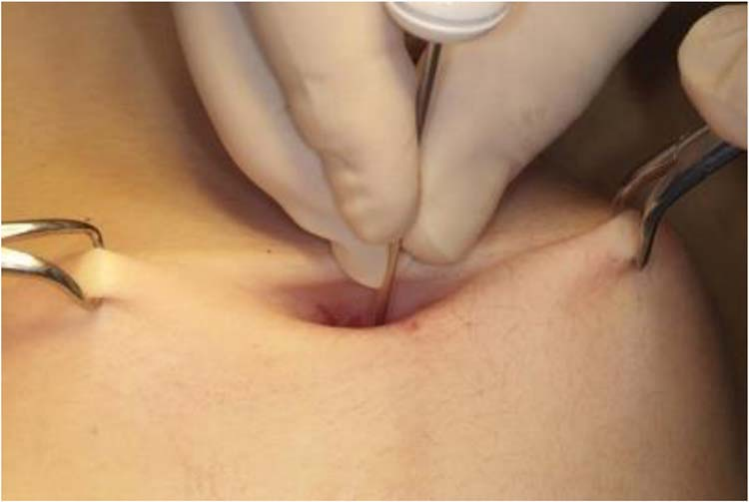
Image of the insertion of the Veress needle.

1. The entire anterior abdominal wall is prepped and draped to ensure asepsis.
2. The midline subumbilical region is usually preferred as the insertion site for the Veress needle as the abdominal wall is thinnest here and all fascial layers fuse into single fascial planes providing a relatively avascular area.
3. A 10 mm incision is made through the skin with a #11 scalpel below the umbilicus to provide an access for the needle (Fig. 1).
4. Laterally on either side of the incision, the skin is elevated with towel clips to distance the anterior abdominal wall from abdominal viscera prior to inserting the needle (Fig. 2).
5. The depth from skin to rectus is measured approximately with the aid of artery forceps inserted vertically through the incision down to rectus sheath. An additional 4 cm is added to this length to get an estimate of the approximate distance from skin to abdominal aorta, which could be at risk at this site (Figs 3 and 4).
6. The Veress needle is assessed for ensure proper functioning prior to insertion.
7. With the Veress needle held in the pen grip, it is inserted perpendicular to the skin directed slightly towards the pelvis, ensuring the needle is not inserted beyond the distance marked (Fig. 5).
8. Two characteristic ‘pops’ representing the fascia and peritoneum are usually felt as the needle passes through the anterior abdominal wall into the intra-abdominal cavity.
9. 10 mls of saline is pushed through the needle and a free flow will indicate correct positioning as the negative intra-abdominal pressure created by lifting the anterior abdominal wall with towel clips ‘sucks in’ the saline.
10. The Veress needle is then connected to an insufflation tubing and CO_2_ insufflation is initially commenced at low flow.
11. If the intra-abdominal pressure remains low, insufflation is increased to high flow until a desired pressure of about 12 mmHg is achieved.
12. After about 1 L of gas has been induced, the Veress needle is advanced a further 5 mm into the abdominal cavity to ensure it does not exit with increasing intra-abdominal pressure.

## DISCUSSION

Employing safe techniques for the induction of pneumoperitoneum is paramount in reducing intra-operative complications such as vascular injury. By approximating the distance from the skin to the abdominal aorta, the surgeon can ensure safe insertion of Veress needle.

## CONFLICT OF INTEREST STATEMENT

None declared.

## FUNDING

None.

## DATA AVAILABILITY

All data are incorporated into the article and its online supplementary material. The data underlying this article are available in the article and in its online supplementary material.
